# Investigating physician self-referral in public hospitals in South East Nigeria: Insights from stakeholders

**DOI:** 10.4102/phcfm.v14i1.3271

**Published:** 2022-10-31

**Authors:** Bartholomew S. Eze, Mari Jones

**Affiliations:** 1 Department of Health Administration and Management, Faculty of Health Sciences and Technology, University of Nigeria, Enugu, Nigeria; 2 Swansea Centre for Health Economics, College of Human and Health Sciences, Swansea University, Swansea, United Kingdom

**Keywords:** dual practice, physician self-referral, patient diversion, physician, public hospital, Nigeria

## Abstract

**Background:**

Physician self-referral occurs where a full-time paid doctor diverts patients from one hospital to another in which he or she has financial interest.

**Aim:**

This study is aimed at investigating the views of service users, physicians and policymakers on physician self-referral practice in public hospitals in Nigeria.

**Setting:**

The study was carried out in Enugu urban area of South East Nigeria.

**Methods:**

A mix of qualitative and quantitative methods was used to collect information from different categories of stakeholders. Service user views were explored through analysis of four focus group discussions involving 26 participants and 407 questionnaires completed with household members who had recently visited a public hospital and then gone to private hospitals. In-depth interviews were completed with 15 public sector doctors not involved in dual practice and eight key policymakers.

**Results:**

Thirty-four of 407 respondents (8.4%) visiting a public hospital were diverted to a private facility associated with the attending public hospital doctor. The research examined age, gender and socio-economic status (SES) as factors that might influence the likelihood of patient diversion. Advice to transfer to a private clinic usually came directly from the doctor involved but might also come from nurses.

**Conclusion:**

Physician self-referral in Nigeria could take different forms. It was found that both direct and indirect forms of diversion exist, suggesting that this is an organised practice in which dual-practice doctors and supporting hospital staff members cooperate. The study recommends, among other things, that service users should be adequately protected from any form of diversion to private practice by the public system employee doctors.

**Contribution:**

This study contributes to understanding the extent and pattern of patient diversion in public hospitals in Nigeria. The findings reveal coordinated tactics for diverting public hospital patients and provide a direction for future research in negative behaviour among healthcare professionals in Nigeria.

## Introduction

Physician self-referral occurs where physicians divert or refer patients to a health care facility that is outside of their primary employment for financial interests. When a physician diverts patients to receive health care services in a facility that the physician owns, leases or stands to gain financially from, the practice is termed physician self-referral.^1,2^ Financial interest could take the form of a commission paid per case referred. Patient transfer could take place between public and private hospitals, private and private hospitals, public and public hospitals or even in a *circular diversion* pattern, where patients diverted to the private sector are sometimes kept for a short or long stay before being referred back to the public sector.^3^ However, this study is primarily concerned with public to private patient transfers.

In Nigeria, dual practice (DP), which implies a practice where health care professionals, particularly medical doctors, engage in concurrent public and private clinical work for remuneration,^3,4^ is permitted for public-sector doctors only in their off-duty hours. The Nigerian Code of Medical Practice regards it as unethical for a registered practitioner on full-time public employment to engage in extramural practice during official duty time. Despite these provisions, some doctors do not seem to adhere to the rule limiting extramural practice during official duty time. There are reports that full-time public sector doctors often operate a 24-h hospital service in their private health care facilities.^5^

Physician self-referral seems common in countries where DP operates, such as Peru, Zimbabwe, Bangladesh, Greece, India, Portugal and the United Kingdom (UK),^6^ and especially in countries with a high demand for private medical services.^2,7^ Physician self-referral also occurs in mixed public and private systems where long public hospital waiting times or restrictions on treatments available with public health service or statutory health insurance funding can be avoided by paying for private care.^8^ The motives for such transfers by health care professionals are oftentimes couched as quality concerns or long wait-times in the public sector, but the self-interest of medical professionals appears to be a key factor.^9^

Arguably, physician self-referrals may have both benefits and challenges for patients and the public system. Those who can afford it may opt out of the public health care system by arranging private health insurance or paying out of pocket, while those who are coerced into diversion without the means to pay may then face serious problems regarding household finances and debt.

Some commentators suggest that self-referral allows ‘cream skimming’, in the sense of diversion of high-value patients and those with less complex conditions from the public system to the private sector.^8^ A research in Indonesia^10^ illustrates how providers may ‘sort’ patients so as to channel the wealthier patients towards their private practices. Other commentators suggest that diversion tends to centre more on cases with less complex conditions, as there is generally more profit throughout from routine cases than extended treatment for admissions that may involve complications. Evidence has shown that physician-owned centres treat proportionately more minor surgical cases and fewer patients with comorbidities than public facilities.^11^ Nevertheless, not all self-referrals should be stigmatised; in some instances, it could be aligned with the best interest of the patient.

One of the criticisms of DP is the indirect means used to divert public clinic patients to the private sector. Physician indirect referral strategies include reducing the quality of service and creating long waiting periods or waiting lists in the public hospitals by not providing timely care.^12^ This is called induced referral,^6^ which means the deliberate reduction of quality in the public sector as a way of encouraging indirect referral, appears to be a growing problem that arises from the central role played by doctors in determining the nature of patient experience. Doctors are able to change their behaviour to differentiate the quality of public and private health care. For instance, evidence from India shows that dual practitioners working in private clinics spent more time with patients, completed more items on a checklist and provided better treatments than they did in their public-sector practice.^13^

A study in Ethiopia^3^ reported a different form of patient diversion whereby public patients initially diverted to the private sector were later redirected back to the public system without receiving proper treatment in the private sector. This has been termed *circular diversion* and refers to a situation where dual practitioners extract maximum payment by admitting patients to their facilities for a short period while lacking the specialist facilities to complete necessary treatments. This predatory behaviour benefits the practitioners financially to the detriment of patients, who are indirectly forced to make additional payments, and of the public system, which must subsidise the more costly phases of treatment.

In Nigeria, there is evidence to suggest that physician self-referral is common among doctors employed in government health facilities. It has been found that over 60% of the public sector doctors earn some extra money from supplementary work outside of their primary employment, whereas 75% would give a higher priority to fee for service jobs.^14^ Furthermore, a more recent study^15^ has clearly shown that government employee doctors in the anglophone West African region, including Nigeria, divert patients to their private practice even when treatment can be provided in the public system. This practice has been attributed to poor pay in the public sector and a coping strategy for many public sector doctors. The lack of solution to this practice has also been blamed for the power wielded by medical professionals in the health system, political connections and implicit immunity to sanctions.^15^ However, while these authors provided information on different types of corruption in the health sector in the region, including patient diversion, the present study offers an in-depth understanding of the practice of physician self-referral using a mixed methods approach that draws on the perceptions of different stakeholders, including service user experience. The study contributes to understanding the practice of physician self-referral in the Nigerian health system by providing information useful to inform debate on what is a pressing public policy issue.

## Methods

### Study design and setting

This study used a convergent mixed methods approach, incorporating both qualitative and quantitative components. Overall, information was gathered from a mix of focus group discussions (FGDs) with service users, in-depth interviews with high-level stakeholders and government doctors, and a household survey of service users. The study was carried out in the Enugu urban area of South East Nigeria. The study area comprises three local government areas (LGAs) and has a high concentration of private and public hospitals, with the majority of government employee doctors located in the area.^16^

### Exploratory descriptive qualitative study

#### Study population, sample size and sampling

The qualitative methods were focus groups and in-depth interviews. Focus groups were completed with a sample of service users and in-depth interviews with key policymakers and non–DP doctors who worked exclusively in the public sector. Four FGDs were carried out, involving 26 service users. The latter were purposively selected to include a balance of men and women (18 years and above) who had first visited a public hospital and then moved to a private hospital in the previous 12 months. Information about their physician self-referral experience was also collected. The researcher used a contact person to recruit and mobilise the participants in this study using the inclusion criteria above. It was helpful to use a contact person who was already familiar with the study area to mobilise the participants and act as a bridge between the participants and the researcher. This approach was meant to avoid any suspicion or fear among the participants. Four focus groups were convened to keep numbers small, in order to encourage discussion while getting the views of a reasonable number of participants. Three FGDs comprised 6–8 persons, while the fourth had four participants.

In-depth interviews were carried out with eight key policymakers and 15 non–DP public-sector doctors, selected using a ‘snowball’ sampling method. Snowball sampling is a technique that allows the existing research subjects to suggest others known to them for recruitment, and it is suitable for conducting exploratory qualitative research, especially with a population that is hard to identify or locate. Therefore, only those stakeholders who have good knowledge of the study objectives were recruited and contacted for interview. The focus groups and in-depth interviews were conducted between 10 October 2015 and 15 November 2015.

### Data collection

An FGD guide was used to elicit information from the participants. Data were collected on their sociodemographic characteristics. The questions asked included their perceptions of DP of medical professionals, experience visiting public hospitals and private practices of public-sector doctors, physician self-referral of public patients and if they have been diverted from public hospitals to private practice. Focus group discussions allow the researcher to understand why the group holds certain views, and the group dynamics could offer insights that an individual interview may not. It is a good method to gain more in-depth information to supplement other primary methods, such as the survey method. The researcher facilitated the FGDs, which were audio-recorded with participants’ permission. The locations were borrowed rooms away from health service settings. Once sufficient participants had consented to take part, the meetings went ahead with all attending and participating. The FGDs lasted approximately for 60 min each.

For other stakeholders, the researcher approached the selected respondents to acquaint them with the study objective and to seek their participation. They were provided with an information sheet and invited to ask questions before signing a consent form. Those who consented were interviewed at an agreed time and location. An interview guide was developed and used to elicit information from the respondents. The sociodemographic characteristics of the respondents were collected, including questions on the current regulation of DP in Nigeria and the implementation challenges, how government doctors are monitored and supervised and their perceptions about physician self-referral. The interviews were audio-recorded with the permission of the respondents.

### Data analysis

The FGDs and interviews were audio-recorded and transcribed verbatim with all identifiers removed. Data were organised using NVivo version 10 (QSR International, Doncaster, Australia). The transcripts were read several times to improve the understanding of the concepts and meanings in the text. The researcher considered familiarisation with the data important as the analysis involved repeated reading of the transcripts with the intent of searching for meanings, patterns and processes.^17^ Themes were generated both deductively, based on ideas from the literature in this area, and inductively, taking into account the new elements emerging from the data. Also, the internal ‘homogeneity’ and external ‘heterogeneity’ of data were considered.^18^ The initial coding was carried out to reveal the meanings and patterns emerging from the data, which helped to generate a provisional list of ideas within the data. A recoding process was applied as themes and subthemes continued to emerge and change. This helped in managing and filtering the data to focus on emergent patterns, refine themes and work out their linkage to relevant concepts. Finally, the major and minor themes were reviewed for relevance. Data within a theme that did not seem to cohere together meaningfully were dropped or separated, while other themes that did not have clear distinctions between them were merged.

### Descriptive survey

#### Study population, sample size and sampling

The quantitative arm involves a household survey intended to illuminate the views of a larger sample of service users and families. The required sample size was calculated using Epi Info 7 (Centers for Disease Control and Prevention, Atlanta, Georgia, United States). The parameters used for the calculation were the population of Enugu South LGA based on the projected 259 000 population by 2015,^19^ power of 80%, confidence limit of 95% and expected frequency of 50%. This suggested that a sample of 384 was required. A pretest of the questionnaire was conducted with 20 respondents, who were not included in the final questionnaire study. To allow for contingencies, a total of 407 valid questionnaires were then completed.

All the selected households included a member who had first visited a public hospital in the previous 12 months and then gone to private hospital. The sample was assembled using a cross-sectional multistage sampling design. In this study, multistage sampling, simple random sampling, systematic sampling and consecutive sampling were utilised at different stages. One LGA was randomly selected from the three LGAs that make up the Enugu urban area. The selected LGA comprises five residential areas, from which two areas were randomly selected for questionnaire administration. A list of eligible streets within the selected residential areas was compiled, and four streets from each were picked randomly. At that stage, systematic random sampling was used to select houses for questionnaire administration, using even or odd numbers. Having selected households, the researcher recruited survey respondents in sequence, based on whether they met the criterion of having visited a public hospital and then gone to a private one in the last 12 months, until the required number of household respondents was achieved. Where a building visited was home to more than one household, consecutive sampling was used to administer the questionnaire to other eligible households occupying the building before moving into the next sampled building.

### Data collection

For the household survey, the questionnaire was administered by a single researcher to minimise misunderstanding of questions or variation in the recording of answers. Data on the sociodemographic characteristics of respondents were obtained, as well as information on the hospital visit experience, including physician self-referral experience. Generally, the respondent was the senior household member present when the researcher arrived to administer the questionnaire. Respondents were guided through the instrument as the researcher asked the questions and filled in their preferred responses, for example, about their hospital visit experiences. The questionnaire data were collected between 28 October 2015 and 05 April 2016, as part of a larger mixed methods study.

### Data analysis

The quantitative data analysis of the household survey was carried out using the Statistical Package for the Social Sciences (SPSS) version 22 (IBM Corporation, Armonk, New York, United States), Stata version 10 (StataCorp LLC, College Station, Texas, United States) and Stat/Transfer software (Circle Systems, Inc., Seattle, Washington, United States). Frequency tables and percentages were generated to represent the sociodemographic characteristics of respondents. Socio-economic status (SES) was disaggregated into quartiles (four groups): Q1 (poorest), Q2 (very poor), Q3 (poor) and Q4 (least poor). Stat/Transfer was used to transfer data from SPSS to Stata. In Stata, the variables of interest – data on household assets, living conditions and household weekly food consumption obtained from the household survey – were used to determine the SES of households using principal component analysis.^20,21^ The variables that were included in the SES index were ownership of key assets such as car, motorcycle, radio, refrigerator, television set, bicycle and grinding machine, together with household weekly food expenditure. This method of determining the SES has been validated and used in previous studies in Nigeria.^22,23^ The SES index created was then transferred back to SPSS for further analysis. The initial aim of this exercise was to examine whether there were systematic differences in the impact of DP on people from different SES groups referred to private facilities. However, this was not pursued as the referral group (*n* = 34) was considered too small to carry out subgroup analysis. Instead, the SES index helped to examine which socio-economic groups were more or less likely to be referred to private practice by dual practitioners.

Descriptive statistics were utilised to describe the sociodemographic characteristics of the study respondents, such as the age and gender profiles of those referred on to private facilities, and to determine providers’ referral patterns. Chi-square tests were conducted to determine if more men than women were self-referred, the SES group most likely to be diverted and the age category most susceptible to diversion. Fisher’s exact test was used when the expected values of the variables fell below 5. Because of the nonparametric nature of the age variable, the Mann–Whitney *U* test was used to determine whether there was a significant difference in median age for the referred and nonreferred groups.

### Ethical consideration

This study adhered to the usual safeguards employed in research on human subjects. Written informed consent was obtained from all participants before completing the questionnaire or participating in interviews, while for the focus groups, oral consent was obtained. Ethical approvals for this study were obtained from a public teaching hospital Committee on Medical and Scientific Research (ref. no. NHREC/05/01/2008B-FWA-00002458-IRB00002323), the State Ministry of Health in South East Nigeria (ref. no. MH/MSD/EC/0181) and the Research Ethics Committee of a university in Wales, United Kingdom (ref. no. 3280415).

## Results

### Sociodemographic characteristics of respondents

Using the basic sociodemographic characteristics recorded in the household survey, the research first examined whether there was evidence that referral varied according to gender, age and educational level. Table 1 shows that 34 of the 407 respondents (8.4%) were referred from the public system to private facilities. This included 18 referred out of 129 men (14.0%) and 16 referred out of 278 women (5.8%). The result shows that sex is statistically significant (*p* < 0.005).

**TABLE 1:** Referrals and nonreferrals and their sociodemographic characteristics.†

Variables	Referred (*n* = 34)		Nonreferred (*n* = 373)		Total (*n* = 407)		*χ* ^2^	Mean	Range	s.d.	Mann–Whitney *U*	*p*
	*n*	%	*n*	%	*n*	%						
Referrals	-	-		-	34	8.4	-	-	-	-	-	-
Nonreferrals	-	-	-	-	373	91.6	-	-	-	-	-	-
**Sex**							7.735					0.005
Male	18	14.0	111	86.0	129	100.0	-	-	-	-	-	-
Female	16	5.8	262	94.2	278	100.0	-	-	-	-	-	-
**Age group (year)**								38.72	19–86	12.49	6500.0	0.808
18–38	22	9.1	221	90.9	243	100.0	-	-	-	-	-	-
39–59	8	6.1	123	93.9	131	100.0	-	-	-	-	-	-
60–80	4	12.9	27	87.1	31	100.0	-	-	-	-	-	-
> 80	0	0	2	100.0	2	100.0	-	-	-	-	-	-
**Highest education level**							8.236					0.243
Primary school	3	7.3	38	92.7	41	100.0	-	-	-	-	-	-
Junior secondary	0	0.0	7	100.0	7	100.0	-	-	-	-	-	-
Senior secondary	10	5.8	161	94.2	171	100.0	-	-	-	-	-	-
Ordinary National Diploma	4	11.8	30	88.2	34	100.0	-	-	-	-	-	-
Higher National Diploma	8	15.7	43	84.3	51	100.0	-	-	-	-	-	-
BSc degree	9	12.7	62	87.3	71	100.0	-	-	-	-	-	-
MSc degree	0	0.0	10	100.0	10	100.0	-	-	-	-	-	-
Other	0	0.0	16	100.0	16	100.0	-	-	-	-	-	-
**Marital status**							3.247					0.354
Currently married	23	7.3	293	92.7	316	100.0	-	-	-	-	-	-
Single	9	13.2	59	86.8	68	100.0	-	-	-	-	-	-
Separated	0	0.0	2	100.0	2	100.0	-	-	-	-	-	-
Widowed	2	100.0	19	90.5	21	100.0	-	-	-	-	-	-
**Occupation**							6.562					0.308
Government worker	0	0.0	30	100.0	30	100.0	-	-	-	-	-	-
Employed in private sector	5	10.4	43	89.6	48	100.0	-	-	-	-	-	-
Self-employed	16	8.4	175	91.6	191	100.0	-	-	-	-	-	-
Artisan	8	11.0	65	89.0	73	100.0	-	-	-	-	-	-
Student	0	0.0	12	100.0	12	100.0	-	-	-	-	-	-
Unemployed	2	5.6	34	94.4	36	100.0	-	-	-	-	-	-
Other	3	17.6	14	82.4	17	100.0	-	-	-	-	-	-

† , The referrals were from public to private hospitals.

Regarding age, 22 of 243 (9.1%) were referred from the 18–38 years age group, while eight of 131 (6.1%) were referred from the 39–59 years age group, and four of 31 (12.9%) were referred from the 60–80 years age group. No one over the age of 80 years was referred. Regarding the educational level of respondents, nine of 71 (12.7%) of those in the highest category who held a university degree were referred, and eight of 51 (15.7%) holding a Higher National Diploma were referred. This compares with 10 of 171 (5.8%) of referred patients who completed secondary education and four of 34 (11.8%) who had achieved an Ordinary National Diploma.

The findings on referrals according to educational level are more complicated and must be treated with caution given the small numbers in each category. Those with the least education are less likely to be referred, while those who have completed senior secondary education (*n* = 10) and those with a Higher National Diploma or bachelor’s degree (*n* = 8 and *n* = 9, respectively) are the categories with most patients transferred to private facilities. Given that the overall percentage of patients referred from all levels is 8.4%, we can see that those with Ordinary National Certificates (11.8% of respondents in this category), Higher National Diplomas (15.7%) and BSc degrees (12.7%) have a higher-than-average probability of referral. It is important to note, however, that there is no straightforward linear relationship between referral and educational level, as none of the 10 respondents with MSc degrees were referred.

The small numbers in each category again make it difficult to draw strong conclusions about occupation and referral. However, the striking findings for further investigation are that government employees are rarely referred (none in our sample) and that the self-employed are well represented among those diverted, accounting for 47% of those referred and the same percentage for those not referred.

**FIGURE 1:**
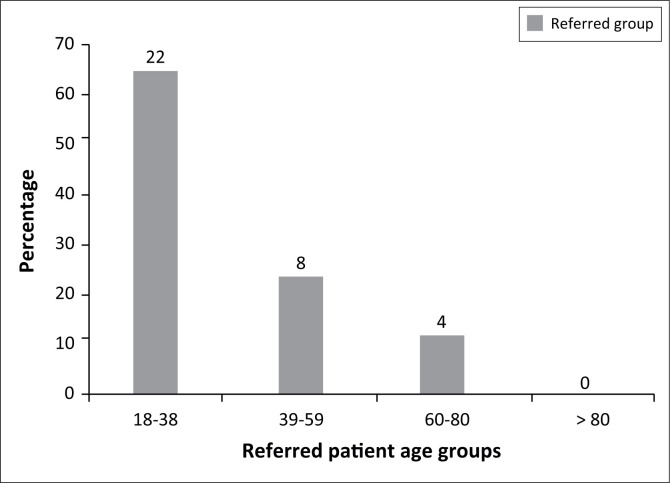
Age groups of patients referred from the public system to private practice (*n* = 34).

Numbers of referrals in different age groups are shown in Figure 1. The largest number of referrals (22 of 34) was in the 18–38 years age group, but the proportion of the age group referred is higher for patients aged 60–80 years (at 12.9% compared with 9.1%), albeit based on a very small number of cases. Eight patients were referred in the age group of 39–59 years, and four in the age group of 60–80 years. Put another way, those aged 18–38 years comprise 64.7% of referrals and 59.2% of nonreferrals, while the corresponding figures for the 39–59 years age group are 23.5% (referrals) and 33.0% (nonreferrals), and for the 60–80 years age group they are 11.8% (referrals) and 7.2% (nonreferrals), respectively. Those over 80 years were not referred. The overall age profile of referred and nonreferred patients is similar, with no significant difference in the median age (*p* = 0.808).

### Socio-economic status of patients referred from public system

Apart from educational level, the study investigated whether SES status index, based on the four categories of poorest (Q1), very poor (Q2), poor (Q3) and least poor (Q4) utilised in the study, influenced the likelihood of referral. Q1–Q4 cover the quartiles of most poor to better off in what is not an affluent area. Table 2 presents data on referred and nonreferred patients and their SES groups. Only 2.0% of the poorest were referred, while the figure was 8.8% for the very poor group, 12.7% for the poor group and 9.9% for the least poor group.

**TABLE 2:** Respondents from different socio-economic status groups referred from the public system to private practice (*n* = 34).

Variable	The poorest		The very poor		The poor		The least poor		Total		*X* ^2^	*p*-value
	*n*	%	*n*	%	*n*	%	*n*	%	*N*	%		
Referred	2	2.0	9	8.8	13	12.7	10	9.9	34	8.4	-	-
Not referred	100	98.0	93	91.2	89	87.3	91	90.1	373	91.6	-	-

**Total**	**102**	**100**	**102**	**100**	**102**	**100**	**101**	**100**	**407**	**100**	**8.360**	**0.04**

### Strategies for facilitating self-referral

**FIGURE 2:**
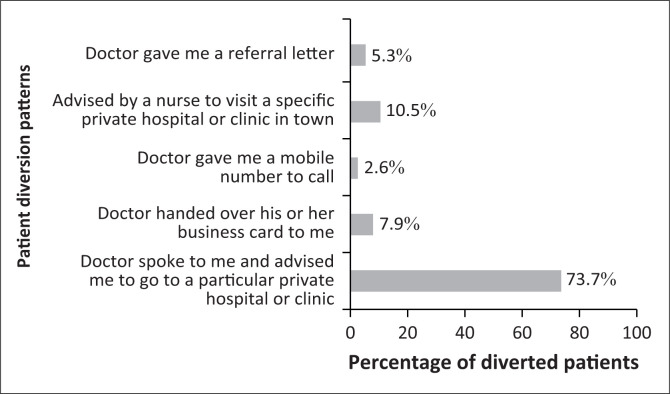
Strategies used by health care providers to refer patients from the public system.

From respondents’ survey responses, referral to a private facility is usually initiated when the doctor suggests this to the patient in a verbal interaction, but many also employ less direct means to persuade the patient to go private. This might include hinting that treatment could be speeded up by ringing a certain telephone number or contacting a clinic on a business card handed to the patient, sending the patient a referral letter and enlisting the help of a nurse to suggest that the patient should request a referral. Figure 2 shows that in over 70% of cases, the topic of transfer to private care was broached in face-to-face meetings by doctors themselves and that in around 10% of cases, it was the nurse who raised the topic.

### Did patients know the private provider works in a government hospital?

**FIGURE 3:**
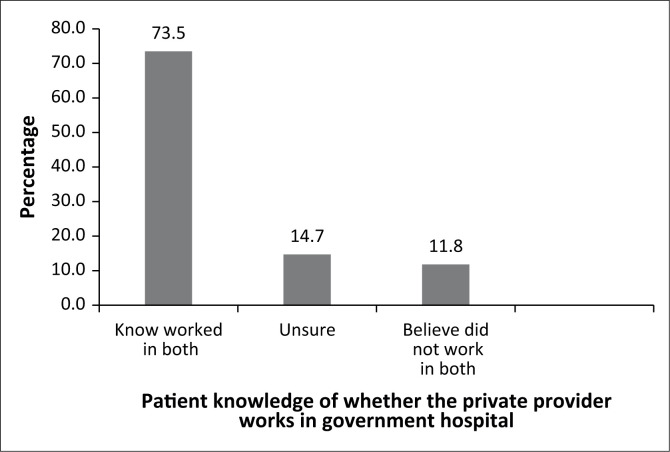
Referred patients’ awareness that the private provider works in a government hospital (*n* = 34).

Figure 3 shows how many of the referred patients were aware that the same provider who treated them in a private hospital also worked in a government hospital. A large majority of referred respondents (73.5%) stated they knew that the doctor worked in both sectors, while 14.7% said they were unsure about this. Only 11.8% said they did not know that the private provider worked in both sectors.

### Stakeholders’ perspectives on physician self-referral

The qualitative data from the focus groups with service users and in-depth interviews conducted with key policymakers and non–DP doctors yielded additional insights on what stakeholders thought of as physician self-referral. A brief account of the key themes emerging from the three respondent groups is presented next.

### Service users’ perspectives on physician self-referral

The majority of service users participating in the focus groups had either experienced or witnessed physician self-referral in the previous 12-month period. Generally, they understood that the dual practitioner self-refers patients to a private facility in which they have a financial interest and may use ‘agents’ such as nurses or other health care workers to assist in this. However, while there was an awareness that referral might be motivated by financial self-interest, this was tolerated because of doubts about the quality of public hospital treatments and a perception that care in private clinics was better. The stories of two patients are recounted in the following FGD extracts:

‘I had been to a government hospital, and they referred me to a private hospital owned by a doctor working in the same government hospital. I stayed the whole day in the government hospital and left without being given attention and came back the following day. When I came the following day, I spoke with one Reverend Sister [*X*], a doctor, who also works in that government hospital. She said that my case would not be treated in that government hospital and that she would direct me to one of their doctors who is a gynaecologist here in this hospital. I left the hospital [*government*] to go to the private hospital where she directed me. When I reached there, the doctor was not there but one of the nurses called him on the phone immediately and explained my condition to the man. And within 10 min he was called, the doctor rushed to his private hospital and opened a file for me without delay. He called his son to take me to where I did the laboratory test, and treatment was commenced immediately, and within six hours I started recovering. But when I went to the government hospital, they said “Eeeeeh, I have never seen this kind of condition in my 21 years of medical practice” [*all FGD participants laughed*]. Maybe he did not study that case in his medical training. But when I came to the private hospital, the doctor commenced work immediately, and before you knew it, he hung my two legs up and started giving me injections. Although that baby later died, I still delivered the baby. He treated me until I was OK. I have had two babies after that one, and there was no problem with my womb.’ (FGD 4, Participant 3, Age 27, Female)

‘I had an experience during my first year at university. I had a severe sickness during this time, and my family took me to a [*public*] tertiary hospital. The money I was charged was not much, but the problem I faced was that of poor service. I spent three days there but did not see the doctor in charge of my case. He did not come to work for these three days. At this time, my legs were swollen up. So my father took me to another government hospital in a neighbouring state. There in that hospital … the same doctor who was supposed to have treated me in the first tertiary hospital I was admitted to was also working in the second hospital I visited. My brother is a medical doctor and he insisted that only that doctor should treat me. What the doctor did was to refer me to where I could take a laboratory test, and after the doctor had seen the test result, he suggested that I should be discharged and sent back to Enugu. When I came down to Enugu, I was admitted to a private hospital and to my great surprise that same doctor owns the private hospital.’ (FGD 2, Partciapnt1, Age 26, Male)

Patients pay extra when they are diverted from the public sector to a private facility. While such transfers may give patients access to experienced consultants and will generally result in more attentive care, it brings a financial burden. Patients generally recognise that referral is more likely to be bound up with profit than altruism but are still prepared to consider it because of perceived quality problems in public hospitals. It might be argued that it is professionals themselves who have brought about the quality gap between the two sectors, but service users see themselves as powerless to change that.

### Non–dual practice doctors’ perspectives on physician self-referral

The researchers had assumed that public hospital doctors not involved in DP would have seen physician self-referral in action but would have no personal stake in defending it or hiding how it was conducted. Our interviews with the 15 doctors approached provided useful insights into what was involved in both direct and indirect diversion, as well as the limitations of the present rules on what is allowed.

Respondents confirmed that both doctors and staff members working with them, mainly nurses, may suggest a referral. The most common scenario is that doctors directly tell patients to go to their private clinics and wait for them:

‘It is like drawing away patients from the public health facility to the private. It is like that. For me, my conscience would not allow me to do that and more so because of my personality. It is insulting to realise that somebody can ask someone, especially in a public health facility, to follow him or her to his or her private setup. Of course, it happens in Nigeria. I would not say that I can name an example, but I know it happens, people telling patients from the public facility to go there and wait for them [*i.e. in their private clinics*].’ (Interview with a General Practitioner, non-DP, July 2015)

Indirect diversion was said to mostly take two forms. Firstly, there is the use of nurses to help doctors to divert patients to their private clinics:

‘Doctors can refer, or they can do that through the nurses. For example, sometimes, after seeing the doctor, they may ask you to come back in 3 days’ time. And the nurse will ask if you asked the doctor for their contact number. But if you did not, the nurse might say, are you a fool? You may not see him at the hospital in the next appointment. You know he is a consultant; go and ask him to tell you about his private hospital. The nurse might give you the consultant’s number, so that it does not look as if it is the doctor that diverted you to his private hospital. When the doctor sees you, he may ask, “What are you doing in my hospital?” Then, the patient will say, “Someone directed me to see you here”. So it is common. But in the teaching hospital, people are always careful about it. It is more common in general and specialist hospitals where the monitoring is not too serious.’ (Interview with a non-DP, Senior Registrar, July 2015)

Secondly, some dual physicians can make the public system difficult for patients to access as a tactic to push them towards their private practices:

‘Because the only way to make patients come to their private clinics is to subtly make it difficult for patients to go to a public hospital, so that way they can drift them toward their private hospitals. But if a doctor consults in other hospitals not owned by him, of course, he is not under much pressure, unlike the pressure he faces when he runs his own private hospital. At the end of the month, whether he sees patients or not, he must pay salaries to his employees, and that may push them to make sure they survive in the market. But if, on the other hand, they are consulting in an established hospital, if they have patients, they go and see them and, if not, they do not lose anything.’ (Interview with a consultant, non-DP, July 2015)

This tactic may seem difficult for patients to detect, especially in a context where health care providers are well trusted and respected.

Respondents explained that although DP has attracted increasing criticism in recent years, the present framework of rules is largely ineffective in stopping it:

‘The circular that was sent to this place is not about the prohibition of DP, but it indicates that government is frowning on it. The situation is bad because medical doctors know the ethics but continue to siphon patients from this health facility [*public hospital*] to their private hospitals. They also have people there working for them. So work is also going on in their own hospitals. But the idea of moving patients from the public hospital is the criminal part of it.’ (Interview with a consultant, non-DP, August 2015)

As the foregoing interview extracts indicate, some doctors deplore self-referral, seeing it as unprofessional behaviour of a kind they would not contemplate. Yet for others, the financial rewards are difficult to resist. Dual practice deprives government hospitals of revenue but is often defended as a way to help patients receive timely treatment. It looks like a double-edged sword and raises the question of whether the diverted patient is satisfied or feels ‘ripped off’ following the private treatment received.

### Policymakers’ views on physician self-referral

Stakeholders in senior positions in federal or state Ministries of Health and hospital administrations appeared well aware of how DP worked. Much of the content of interviews with this group again touched on how doctors might either make direct approaches to patients or use hospital staff members to arrange diversion on their behalf. This study’s informants explained how doctors use a variety of pretexts to push patients towards private care. They may, for example, dishonestly claim that all the public beds are already taken and that the urgency of the patient’s condition means they must pay to get seen:

‘There was a senior medical officer who hid under the guise that all available bed spaces are occupied and that they cannot admit a patient on the floor. And this patient was an eclamptic patient, and an eclamptic patient would have convulsions … high blood pressure, and in that condition the mother and the foetus are at risk. So this patient was taken to this senior medical officer’s private facility, and the patient died on the operating table.’ (Interview with a senior administrator, policy maker, June 2015)

The senior stakeholders confirmed the claim of non–DP doctors and service users that DP doctors use agents as an unobtrusive way to divert patients to their private practice. Interestingly, one informant said that both health care and non–health care staff may get involved in this practice and be paid for diverting patients to the doctor’s private facility:

‘I remember when I was doing my house job in a general hospital in State [*X*]. They posted me to the Obs and Gynae Unit of the hospital. Of course, many of these deliveries and complications would come up at night, and I had my senior medical officer who was deputising for the chief consultant who was not available that night. Right from the gate of the hospital, you could see the gate men scouting for patients to refer to the doctor’s private hospital so that they could get some commission.’ (Interview with a senior administrator, regulatory council, April 2016)

As several informants indicated, middle-ranking doctors starting their own private hospitals face continual pressure to generate revenue to support their staff and other costs, so that maintaining a good flow of diverted cases, often in competition with other doctors who own rival private facilities, becomes a major preoccupation. In this situation, payment of ‘commissions’ is far from uncommon.

Although doctors can often find reasons to justify self-referral, the key policymakers interviewed generally took a negative view of the practice. They suggested that diversion not only robs public hospitals of revenue but also increases the cost of care to patients and sometimes results in negative outcomes. From the standpoint of the critical administrators, dual practitioners’ private facilities are not equal partners operating alongside the state sector, but entities that feed off the public hospital and deplete its resources and ability to provide quality care:

‘A typical example is when you go to private clinic [*X*] – where Dr [*Y*] and company were doing private practice. When they were working at the government hospital [*A*] – in Enugu, the place [*the private clinic*] was booming, but when they retired, their source of patient supply [*government hospital*] dried up. When they were working there, you would never see them at the government hospital; they were busy diverting patients from the hospital to their private clinic. But when they retired from the government hospital there was no more source of diversion. This is a typical case study that when you study them you will then be laughing. If you go there now, it is a shadow of itself.’ (Interview with a senior administrator, policy maker, July 2015)

The difficulty in Nigeria is that many senior figures within the medical establishment are themselves owners of private medical facilities, so that the critics of DP interviewed in the present study are probably not representative of opinion in the professional bodies or the Ministry of Health. The lack of firm support for stricter regulation of DP in high-level policy circles means that reform of the present arrangements remains a distant prospect.

## Discussion

From this analysis of what is admittedly a small sample of diverted patients, the modal patient subject to self-referral is a married man of working age, in self-employment, with a higher-than-average level of education and from one of the two higher-quartile SES groups in the study. Therefore, caution must be made in summarising the results because of the small subsample of the referral group, so the patterns discernible relate only to the sample rather than to the wider population.

The study found that men were more likely than women to be diverted. More men (14.0%) than women (5.8%) were diverted to the private sector (*p* = 0.005). This is in line with Nigerian cultural norms that cast men in more economically independent roles than women, and it probably reflects a perception on the part of DP doctors that men will be better able to pay for private care. In Nigerian society, women’s ability to pay medical bills will in many cases be dependent on the support of a male partner or relative. A study of public-on-private DP in Ethiopia recorded a higher number of men than women admitted in zonal hospitals but did not show which gender was more likely to be referred.^3^ The present study suggests that men on average are more likely to be transferred to a private facility by dual practitioners.

By far the largest group of patients referred was found in the 18–38 years age group (22 of 34). However, what seems surprising from the study is that a small number of patients aged 60–80 years (four patients) had a higher mean likelihood of referral (although this result is not statistically significant, *p* = 0.808). It seems likely that working age patients are in fact the group most likely to transfer to private care, but there are grounds for believing that older patients may also provide a flow of patients for diversion. In Nigeria, many elderly persons do not visit the hospital except at a crisis point. Social care provision in Nigeria is minimal and does not cater for the elderly. In times of crisis, immediate intervention is necessary but may not be available in the public system because of bureaucracy and delays, so treatment in a private facility may be the only option. This is probably the type of case most likely to be attractive for self-referral in public hospitals. The investigator could find no empirical study that examined the age profile of diverted patients. Past studies discuss diversion of patients but without this level of detail.^3,6^

It was found that better-off patients from Q3 and Q4 were diverted more than those from Q1 and Q2 (*p* = 0.04). As diversion of patients is probably motivated by the financial benefit coming to the doctor, there may be no incentive to divert the poorer patients who lack the ability to pay for services. It can be deduced that there may be an element of cream-skimming in the selection of less poor patients for referral to private facilities, and this kind of pattern has been reported in earlier studies.^8,10^

The tactics used to divert patients are often direct but could also be subtle and devious. Apart from direct verbal advice from the doctor, there are various other ways of influencing patients through letters or passing on business cards. Where the physician advises a referral, this might be couched as professional advice about the route to the best treatment, and in some cases it may genuinely be in the interest of the patient (or not). It also sounds suspicious to refer patients from government tertiary hospitals with a team of experts and relatively better equipment to a private one, often a solo practice in town.

Indirect diversion of patients also takes different forms. Nurses may be used as mediators to push patients towards visiting the consultant’s private practice. This is often cleverly done when the nurse indirectly prods the patient to take the consultant’s telephone number and suggests they arrange to see him in a private clinic as he might not be available for their next appointment at the public hospital. Another form of indirect diversion is to make things difficult by creating delays or hurdles for patients in the public hospital so that some patients themselves ask if they can see the consultant privately. At other times, the mere nonavailability of a sought-after consultant, who makes no effort to make himself accessible within the public sector, will be enough to move some patients to ask if they can be seen in his private practice. Indirect diversion has not been described in detail in the literature on DP. The authors found only one previous study that has touched briefly on how physician ownership of a facility can lead to indirect inducements that channelled patients towards using it.^24^

Unnecessary referrals often occur because of a classic principal–agent problem associated with asymmetry of information available to health care provider and patient. Patients believe that the doctors and nurses treating them are disinterested agents who are working to protect their interests, but this is often not the case. Diversion increases the cost of care for service users and reduces the user fees flowing into the public system. The result of diversion may be that a patient who would have received low-cost treatment in a public facility now faces a hefty bill for private services. In some cases, the quality of care may be worse rather than better. Patient diversion has been reported in several different national settings.^3,6,10^ The diversion rate seems lower in this study than that found in the Ethiopian research.^3^ The referral rate in the latter study was 19.2%, whereas the present study found an overall rate of 8.4%, rising to around 10% if an adjustment is made to compensate for the skewing towards female patients in the sample. One of the major limitations of the present study is that the household sample in this study includes substantially more women than men, and this skewing reflects who was likely to be at home when the researcher called. The skewing partly explains the low (8.4%) number of referral cases so that with a sample of 50/50 men and women, this figure could be above 10%. One possible solution will be to recruit an equal number of male and female respondents to see the extent of physician self-referral in each of them. Again, interviewing non–DP doctors may have introduced bias as they could have been negatively predisposed to comment about their professional colleagues engaged in DP. It is also recommended that future research should consider using a dynamic economic model that can clearly identify how patients are transferred from the public system to private practice. This is beyond the scope of this study.

This study further recommends the following. Firstly, service users should be adequately protected from any form of diversion to private practice by the public system employee doctors. Many of the diverted patients could find it difficult to pay private health care bills except by borrowing. Therefore, the public system should take the responsibility for protecting service users from diversion. Secondly, the Patients’ Bill of Rights document could be reviewed to give patients more power to report any diversion attempt to private practice by health care providers without victimisation. Unfortunately, the implementation of patients’ rights in Nigeria is weak and the majority of patients have no ‘voice’ to report abuses. Also, the institutional mechanism that acts as ‘patient voice’ in Nigeria is too bureaucratic, with weak implementation and sanction. Thirdly, there is an urgent need to tackle absences and late coming to work in Nigerian public hospitals. Absences and late coming to work by consultants and senior medical doctors could be a subtle way to make patients accept diversion without questioning, casting doubts about the quality of treatments in public hospital and the perception that care in private clinics is better. Therefore, an intramural approach where public doctors at senior level could be allowed to treat their private patients within the public facility where they work merits consideration. This approach would raise doctors’ income to a reasonable level. The hospital management can agree with them on the number of private patients they can admit at a given time. The public system could benefit from this arrangement by curbing extramural practice among government doctors.

## Conclusion

Diversion of patients to private practice seems to be a common occurrence among public sector doctors in Nigeria. It takes different forms and affects patients in different ways. The study found that both direct and indirect forms of diversion exist, suggesting that this is an organised practice in which DP doctors and supporting hospital staff members cooperate. Commentators have identified a need for evidence on the prevalence and effects of DP on health care systems, especially in developing countries.^25^ The present study makes a start in mapping out the extent and pattern of diversion in Nigeria. Respondents generally agreed that the present professional regulatory arrangements are ineffective in controlling what appears to them a problematic practice.

## References

[1] Hughes DR, Behargavan M, Sunshine JH. Imaging self-referral associated with higher costs and limited impact on duration of illness. Health Aff. 2010;29(12):2244–2251. 10.1377/hlthaff.2010.0413 21134926

[2] McPake B, Russo G, Hipgrave D, Hort K, Campbell J. Implications of dual practice for universal health coverage. Bull World Health Organ. 2016;94:142–146. 10.2471/BLT.14.151894 26908963 PMC4750430

[3] Abera GG, Alemayehu YK, Henry J. Public-on-private dual practice among physicians in public hospitals of Tigray National Regional State, North Ethiopia: Perspectives of physicians, patients and managers. BMC Health Serv Res. 2017;17:713. 10.1186/s12913-017-2701-6 29126453 PMC5681802

[4] Do N, Do YK. Dual practice of public hospital physicians in Vietnam. Health Policy Plan. 2018;33(8):898–905. 10.1093/heapol/czy075 30289510

[5] Osuagu EM. Ethics and medico legal aspects of medical practice. Enugu: Jaron Industries Ltd.; 2010.

[6] Garcia-Prado A, Gonzalez P. Whom do physicians work for? An analysis of dual practice in the health sector. J Health Polit Policy Law. 2011;36(2):266–294. 10.1215/03616878-1222721 21543706

[7] Rispel LC, Blaauw D. The health system consequences of agency nursing and moonlighting in South Africa. Glob Health Action. 2015;8(1):26683. 10.3402/gha.v8.26683 25971400 PMC4430689

[8] Cheng TC, Haisken-DeNew JP, Yong J. Cream skimming and hospital transfers in a mixed public-private system. Soc Sci Med. 2015;132:156–164. 10.1016/j.socscimed.2015.03.035 25813730

[9] Humphrey C, Russell J. Motivation and values of hospital consultants in south-east England who work in the national health service and do private practice. Soc Sci Med. 2004;59(6):1241–1250. 10.1016/j.socscimed.2003.12.019 15210095

[10] Rokx C, Giles J, Satriawan E, Marzoeki P, Harimurti P, Yavuz E. New insights into the provision of health services in Indonesia: A health workforce study. Washington, DC: The World Bank; 2010.

[11] Mitchell JM. Effects of physician-owned limited-service hospitals: Evidence from Arizona. Health Aff (Millwood). 2005;24(suppl_1):W5-481–490. 10.1377/hlthaff.W5.481 16249249

[12] Kiwanuka SN, Kinengyere AA, Nalwadda C, Ssengooba F, Okui O, Pariyo GW. Effects of interventions to manage dual practice. Cochrane Database Syst Rev. 2010;(3):CD008405. 10.1002/14651858.CD008405 PMC6791302 21735429

[13] Das J, Holla A, Mohpal A. Quality and accountability in health care delivery: Audit-study evidence from primary care in India. Am Econ Rev. 2016;106(12):3765–3799. 10.1257/aer.20151138 29553219

[14] Akwataghibe N, Samaranayake D, Lemiere C, Dieleman M. Assessing health workers’ revenues and coping strategies in Nigeria – A mixed methods study. BMC Health Serv Res. 2013;13(387):2–17. 10.1186/1472-6963-13-387 24093219 PMC3853328

[15] Onwujekwe O, Agwu P, Orjiakor C, et al. Corruption in Anglophone West Africa health systems: A systematic review of its different variants and the factors that sustain them. Health Policy Plan. 2019;34(7):529–543. 10.1093/heapol/czz070 31377775 PMC6788210

[16] Eze BS. Dual practice of medical professionals in public hospitals in south-eastern Nigeria: An economic and policy analysis. Swansea University, 2019, Swansea.

[17] Saldana J. The coding manual for qualitative researchers. 2nd ed. London: Sage; 2013.

[18] Braun V, Clarke V. Using thematic analysis in psychology. Q Res Psychol. 2006;3(2):77–101. 10.1191/1478088706qp063oa

[19] National Population Commission. National census. Abuja: The National Population Commission; 2006.

[20] Filmer D, Pritchett L. Estimating wealth effects without expenditure data–or tears: An application to educational enrollments in states of India. Demography. 2001;38(1):115–132. 10.1353/dem.2001.0003 11227840

[21] Onwujekwe OE, Ojukwu J, Ezumah N, Uzochukwu B, Dike N, Eze S. Socio-economic differences in preferences and willingness to pay for different providers of malaria treatment in southeast Nigeria. *Am J Trop Med Hyg*,. 2006;75(3):421–429. 16968915

[22] Eze BS, Ochonma OG, Ajuba M, Obikeze E. Contextual access challenges to the antiretroviral therapy for HIV-infected persons in southeast Nigeria. Am J Res Commun. 2015;3(11):91–110.

[23] Okoronkwo IL, Onwujekwe EO, Ani FO. The long walk to universal health coverage: Patterns of inequities in the use of primary healthcare services in Enugu, Southeast Nigeria. BMC Health Serv Res. 2014;14:132. 10.1186/1472-6963-14-132 24655898 PMC3984026

[24] Mitchell JM, Sass TM. Physician ownership of ancillary services: Indirect demand inducement or quality assurance. J Health Econ. 1995;14(14):263–289. 10.1016/0167-6296(95)00003-Z 10145136

[25] Berman P, Cuizon D. Multiple public-private job holding of health care providers in developing countries: An exploration of theory and evidence. London: British Government’s Department for International Development (DFID); 2004.

